# Oxygen as a drug and scarce commodity: Do we use it rationally?

**DOI:** 10.4102/safp.v64i1.5544

**Published:** 2022-09-21

**Authors:** Linda Groenewald, Lurika Faber, Jean-Pierre Fourie, Cornelius J. Oosthuizen, Miécke Müller, Kayla van der Westhuizen, Dian D. Kapp, Righard Swanepoel, Hanneke Brits

**Affiliations:** 1 Department of Family Medicine, Faculty of Health Sciences, School of Clinical Medicine, University of the Free State, Bloemfontein, South Africa

**Keywords:** oxygen therapy, COVID-19, prescription, medical drug, wastage

## Abstract

**Background:**

Medical grade oxygen is classified as a drug and needs to be prescribed by a qualified healthcare professional. Oxygen therapy is prescribed to people who cannot maintain normal blood oxygen saturation while breathing atmospheric air. The coronavirus disease 2019 (COVID-19) pandemic highlighted the importance of the rational use of this scarce commodity. This study investigated oxygen therapy practices in adult ward patients.

**Methods:**

A cross-sectional study design with an analytical component was used in the adults wards at a National District Hospital and the Pelonomi Academic Hospital in Bloemfontein. Data were collected from patient files, interviews and oxygen measurements of adult patients that received oxygen.

**Results:**

One hundred and fifteen patients were included in the study, of whom 47.0% received oxygen without an oxygen prescription. Around 62.3% of the patients with prescriptions did not receive oxygen as prescribed. The prescriptions and oxygen administration for COVID-19 patients were better than for non–COVID-19 patients. A quarter of the patients possibly received oxygen therapy unnecessarily.

**Conclusion:**

Poor oxygen therapy practices were identified, including prescription errors, oxygen administration errors and oxygen wastage. A protocol should be developed and implemented for the prescription and administration of oxygen therapy. Training should occur to prevent oxygen wastage.

**Contribution:**

This study highlighted poor oxygen practices and prescriptions, as well as oxygen wastage in the absence of local oxygen therapy guidelines.

## Introduction

Oxygen is a colourless gas present in air and water. It comprises 21% of the Earth’s atmosphere and is one of the most prevalent elements on the planet.^1^ Humans absorb oxygen via the respiratory system, which then plays an important role in cellular respiration – a process that degrades proteins, fats and sugars and produces the energy that living organisms need to function.^2,3^ The primary source of oxygen in the atmosphere is plants (via photosynthesis) and phytoplankton.^4^ It is also possible to produce oxygen commercially using cryogenic distillation, different chemical reactions and electrolysis.^1,2^

Oxygen therapy is prescribed to people with medical conditions that render them unable to consume adequate oxygen through normal breathing.^2,5^ Medical grade oxygen is classified as a drug given to patients in a clinical setting to treat various medical conditions^2,5^ and needs to be prescribed by a qualified physician who has in-depth knowledge related to oxygen therapy.^6,7^

A patient needs oxygen therapy when the measured oxygen is below a certain level (hypoxemia).^2^ This level can be measured directly from blood or indirectly by using a pulse oximeter (a peripheral blood oxygen saturation below 92%).^8^ Hyperoxemia must also be avoided through continuous adjustments,^2^ as high blood oxygen levels can lead to the following complications^9^:

- hypoventilation or apnoea when hypoxic patients suddenly receive high oxygen concentrations
- atelectasis of the lungs due to displacement of nitrogen gas by high concentrations of oxygen
- damage to cells, proteins and membranes caused by unnecessary or high oxygen concentrations over a long period.

Patients can receive oxygen via different devices, including facemasks, nasal cannulas and endotracheal tubes.^10^

The coronavirus disease 2019 (COVID-19) pandemic highlighted that medical grade oxygen should be considered a scarcity in Africa. It is not always available or affordable for all facilities.^5,7,11^ Ugandan researchers found that even before the COVID-19 pandemic, many African countries battled with shortages of oxygen and oxygen equipment.^12^ The additional need for oxygen was emphasised in a study from China, where 41.3% of admitted COVID-19 patients needed supplemental oxygen.^13^ The need for the rational use of oxygen was also highlighted in a study done in sub-Saharan Africa, showing the higher demand for oxygen during the COVID-19 pandemic.^14^

This study aimed to investigate oxygen therapy practices in general adult wards in two training hospitals. The first objective was to determine the percentage of patients who received oxygen according to their prescription; the second was to compare oxygen prescriptions and administration between COVID-19 positive and non-COVID patients; the third was to look at possible unnecessary oxygen use and wastage.

## Methodology

### Study design

A cross-sectional study design with an analytical component was used.

### Target population and sample size

The target population was nonemergent oxygen-receiving adult patients in the general adult medical wards at the Bloemfontein academic training facilities. The study sample consisted of patients adhering to the inclusion criteria, admitted to Pelonomi Academic and National District Hospitals between October 2020 and mid-December 2020, on the days the researchers collected data. Convenience sampling was used to determine the facilities, the study period and the data collection dates. Considering the average number of patients receiving oxygen and the period of data collection, a sample size of approximately 200 was set; however, the global COVID-19 pandemic and the South African lockdown regulations imposed significant time and logistical constraints on the execution of this study. The pandemic also created an opportunity to include COVID-19 patients in the study. Most COVID-19 positive patients at the training facilities met the inclusion criteria for this study.

The inclusion criteria were 18 years and older patients, who were receiving oxygen therapy in general adult medical wards at Pelonomi Academic Hospital and National District Hospital, irrespective of their COVID-19 status. Only patients who received oxygen delivered via facemasks and nasal cannulas were included.

The exclusion criteria were patients in the preoperative and postoperative wards, the intensive care unit and the emergency rooms (i.e. all patients who were not admitted to the general medical wards and COVID-19 wards) and those unable to give informed consent.

### Data collection

The data collected were used to evaluate the oxygen therapy practices at the study sites. The records included demographic data of the patients, prescription of and administration of oxygen, patient vital signs and actual patient compliance to the prescribed oxygen therapy regimen.

The researchers were divided into two groups of four people each: one group went to Pelonomi Academic Hospital and the other group to National District Hospital. These two groups alternated between the hospitals weekly from October to mid-December 2020 to collect the relevant data.

The patient’s vital signs, including heart rate, respiratory rate and blood oxygen saturation, were recorded. A pulse oximeter was used to measure the peripheral blood oxygen saturation. A standard pulse oximeter, approved for medical use by the Food and Drug Administration (FDA), was used according to the manufacturer’s instructions. The product information leaflet states that the meter’s accuracy was within 2% – 3% of the measured blood oxygen levels. A less than 92% saturation reading indicated that the patient needed more oxygen. In comparison, a reading of more than ≥ 97% showed that the patient did not need oxygen or had possibly received too much oxygen.

The researchers compared the oxygen therapy received by the patient with the oxygen therapy prescribed in terms of the oxygen administration apparatus and the flow rate of oxygen. When these two aspects did not correspond with the prescription as predefined in the study protocol, an incorrect method of oxygen therapy was logged. The oxygen flow rate was recorded from the oxygen flow meter beside the patient’s bed and was also compared to the patient’s oxygen prescription and recorded.

A structured interview was then conducted with the patient regarding their adherence to the oxygen therapy regimen. The researchers examined the patients to identify any factors that could have influenced the blood oxygen saturation measurement. These included tremors, cold extremities and the presence of nail polish, among others.

After all the required information was obtained from the patient, the patient file was marked with a blue X to ensure that this patient would not be included in the study again. None of the included patients was admitted more than once.

Owing to the regulations surrounding the COVID-19 pandemic, research assistants with complete personal protective equipment collected the data of COVID-19 patients.

### Pilot study

A pilot study to assess the feasibility of the main study was performed at National District Hospital. Five patients from National District Hospital who adhered to the inclusion criteria were included in the pilot study.

The pilot study revealed some shortcomings with the original datasheet, such as the order of questions and the possibility to include COVID-19 patients. The data from the pilot study were not included in the main study. The pilot study was initially scheduled to be conducted during April 2020. Still, because of the COVID-19 pandemic, the timeline could not be adhered to, and the pilot study was only conducted during August 2020. An amended protocol that included COVID-19 patients was submitted and approved.

### Data analysis

The researchers collected all information on data collection sheets and transferred it to a Microsoft Excel spreadsheet. The Department of Biostatistics at the University of the Free State assisted with data analysis using the statistical analysis software SAS 9.4. The results were summarised by frequencies and percentages (categorical variables), and for comparisons, a *p*-value of < 0.05 was considered statistically significant.

## Results

### Demographics

In total, 115 patients were included in the study. Two-thirds (67.0%) were recruited from the National District Hospital and 33.0% from Pelonomi Academic hospital. Female patients represented 55.7% of the study population. The age distribution was between 21 and 96 years, with a median age of 57 years. Most patients were diagnosed with respiratory or cardiovascular disorders (80.0%), followed by gastrointestinal conditions, endocrine disorders and patients for palliative care. The COVID-19 status of 87.0% of patients was known, while 13.0% were still under investigation. Almost a quarter (23.0%) of the patients with results tested positive for COVID-19.

### Adherence to oxygen prescriptions

Of the patients who received oxygen, 61 (53.0%) had a basic oxygen prescription indicating the device and flow rate. For these patients with prescriptions, the most common oxygen administration apparatus prescribed was oxygen facemasks (88.5%), followed by non-rebreather masks (6.7%) and nasal cannulas (3.2%). One patient (1.6%) received high-flow nasal oxygen.

**FIGURE 1:**
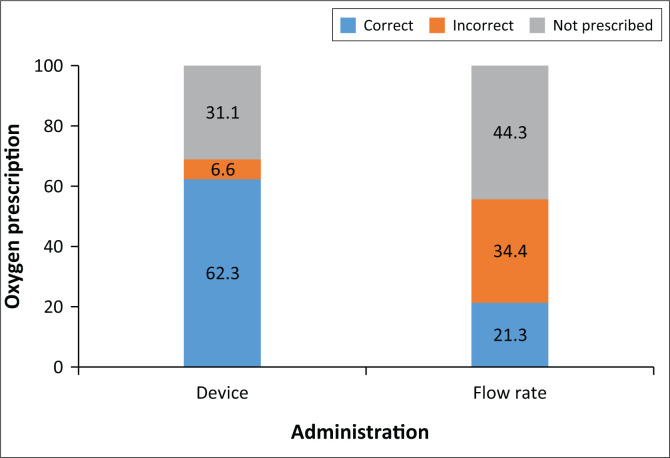
Oxygen prescription versus administration.

When comparing the oxygen prescription with the oxygen received, 62.3% of patients received oxygen through the correct device and 21.3% received the correct flow rate as prescribed. In Figure 1, this comparison is illustrated.

In total, 37.7% of patients received oxygen with both the correct device and flow rate, as prescribed.

### COVID-19 patients

**FIGURE 2:**
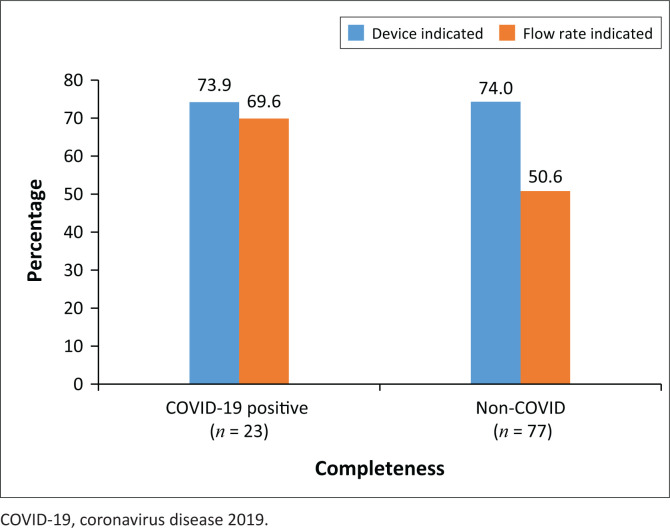
Completeness of COVID-19 versus non–COVID-19 prescriptions (*n* = 100).

Coronavirus disease 2019 test results were available for 100 (of 115) patients. The completeness of oxygen prescriptions for COVID-19 positive patients compared to non–COVID-19 patients is displayed in Figure 2. In both groups, the device was prescribed in 74.0% of cases. The indication of the flow rate on the prescriptions was significantly better (*p* = 0.0486) for COVID-19 patients compared with non–COVID-19 patients.

### Oxygen usage

At both facilities, 70.5% of patients indicated that they received appropriate information on how to use their oxygen. In total, 62.6% of patients mentioned that they removed their oxygen from time to time when they were not supposed to. Of these, 26.4% removed the oxygen five or more times a day without closing the oxygen delivery system. The oxygen saturation of patients was measured while they were on oxygen. In 25.2% of patients, the oxygen saturation was ≥ 97%.

## Discussion

Owing to the COVID-19 pandemic, only 115 patients were included in this study. The pandemic limited the time for data collection and the available patients but provided the opportunity to include COVID-19 patients.

More female patients (55.7%) than male patients were included in this study. A report published in South Africa illustrates that this distribution corresponds with the 2020 COVID-19 gender distribution in South Africa, where 57.8% of COVID-19 positive adults were female.^15^

Hypoxaemia is the number one indication for oxygen therapy.^16,17,18^ Diseases that often cause hypoxemia include heart failure, cardiac rhythm abnormalities, respiratory distress syndrome, asthma and lower respiratory infections.^19^ Similarly, most patients from this study who received oxygen were diagnosed with cardiac and respiratory conditions.

Oxygen is a drug and should be prescribed according to specific clinical guidelines. The basic requirements for an oxygen prescription include the type of oxygen administration apparatus, the flow rate, the duration of the oxygen therapy and the target blood oxygen saturation level.^20^ Currently, there are no guidelines for the prescription of oxygen in South African state-owned hospitals. Therefore, doctors working in these hospitals may not know how to prescribe oxygen therapy correctly. Forty-seven percent of the patients who received oxygen in our study did not have a basic oxygen prescription. Almost a third of patients who received oxygen did not have a prescription indicating the relevant oxygen administration apparatus, while the flow rate was not indicated on more than 40% of prescriptions.

These poor oxygen prescription practices are not unique to this study or South Africa. A study conducted in Saudi Arabia found that only 85.0% of patients that received oxygen in the general male wards had a prescription for oxygen. Their results indicated the flow rate on 67.0% of prescriptions and the target blood oxygen saturation in only 30.0%.^21^ A British study found that 42.5% of patients received oxygen without an oxygen prescription. A report from the Waikato Hospital in New Zealand found that only 51.7% of patients had a correct oxygen prescription.^17,22^ In a Portuguese study, the delivery device was not prescribed in 20.0% of the cases.^23^

The majority (56.5%) of patients had a prescription for facemask oxygen, and very few (3.5%) patients had a prescription for a nasal cannula. Only one patient had a prescription for a high-flow nasal cannula. In direct contrast with these findings, a study done in New Zealand concluded that patients suffering from respiratory diseases in general wards need to be prescribed high-flow nasal cannulas to improve their respiratory function.^24^ The majority of patients in our study were diagnosed with respiratory or cardiac disorders but were prescribed simple oxygen facemasks and not high-flow nasal cannulas as in the New Zealand study. This may be because high-flow nasal cannulas were not used in general adult wards, as oxygen delivery devices in our setting before the COVID-19 pandemic or was not indicated.

Although oxygen therapy was prescribed incorrectly for many patients, some patients for whom oxygen was prescribed also did not receive the oxygen therapy as indicated on their prescription. Only 37.7% of patients received the correct method of oxygen therapy (flow rate and oxygen administration apparatus) as indicated on their prescription. Such poor implementation was also seen in a study done in England, which found that out of all patients receiving oxygen therapy, only 16.0% matched the prescription.^1^ This is alarming, given that oxygen is classified as a drug.^5^ Therefore, oxygen therapy needs to be administered with the same vigilance and caution as any other medical drug.

It has been shown that high-flow nasal oxygen therapy improved the survival rate of COVID-19 patients that were admitted to the Groote Schuur and Tygerberg Hospitals in South Africa.^20^ The results from the Wuhan study further supports the use of high-flow nasal cannulas in the successful management and treatment of COVID-19.^25^ The reasons for the low incidence of high-flow nasal oxygen prescriptions in a COVID-19 setting are not entirely clear. It may be that more severe COVID-19 patients were admitted in COVID high care settings and not in general wards. In Figure 2, it can be seen that the oxygen prescriptions of COVID-19 patients were more accurate in terms of the flow rate and the oxygen administration apparatus. The availability and regular update of COVID-19 treatment protocols may contribute to better oxygen practices in these patients.

The liberal use of oxygen could possibly become harmful above a 94% – 96% blood oxygen saturation.^16^ It is concerning that 25% of the patients in the target population received oxygen therapy despite having a blood oxygen saturation of ≥ 97% without adjustment. A reason for the unnecessary continuation of oxygen therapy may be that a target oxygen saturation was not prescribed, as is necessary for a correct oxygen prescription.^20^

Oxygen delivery devices are intrusive and that patients often remove their oxygen to perform basic tasks such as eating and sleeping.^5^ In this study, 62.6% of patients indicated that they removed their oxygen supply when it needed to remain on. It is important to note that the hospital uses a central system to deliver oxygen continuously to the wards. Each time a patient removes their oxygen device, oxygen continues to flow and is wasted unless closed. The oxygen wastage can be calculated from the number of times these devices are removed, the number of patients that removed their devices, the period they take the oxygen device off and the average oxygen flow rate. If a patient takes off the mask for only 30 min a day at a flow rate of 8 L/min, that patient wasted 240 L of oxygen.

## Conclusion

The researchers noted poor oxygen therapy practices in the adult wards of the study sites, as only 53.0% of the patients who received oxygen had a prescription for oxygen. Oxygen prescription practices were also poor, lacking information regarding the oxygen device and required flow rate. Just over a third received oxygen as prescribed. Oxygen therapy practices were significantly better for COVID-19 patients than non-COVID patients. Although not quantified, it can be deduced that oxygen was used unnecessarily in many patients.

Poor oxygen therapy practices may be due to the lack of guidelines for oxygen therapy in South Africa, the nonrecognition of oxygen as a medical drug and inadequate training regarding oxygen prescriptions.

### Recommendations

We recommend that a protocol is developed and implemented for the prescription and administration of oxygen therapy.

### Limitations

The study was limited because of a smaller sample size than was intended as a result of the COVID-19 pandemic. Future studies should consider expanding the study population to include a wider variety of South African hospitals over a longer study period. This study also made use of convenience sampling, which should not be considered for a larger project. Although the FDA-approved pulse oximeter was used according to the manufacturer’s instructions, the oximeter was not calibrated before each use.
